# Handgrip weakness is associated with motor cortex atrophy in rheumatoid arthritis: a cross-sectional study with a hand exercise intervention

**DOI:** 10.1186/s12916-026-04956-z

**Published:** 2026-05-26

**Authors:** Amanda L Gustafsson, Rolf A Heckemann, Malin C Erlandsson, Victoria ME Lundgren, Zhao Dai, Sofia T Silfverswärd, Rille Pullerits, Maria I Bokarewa, Caroline Wasén

**Affiliations:** 1 Department of Rheumatology and Inflammation Research, Institute of Medicine, Sahlgrenska Academy, University of Gothenburg, Gothenburg, Sweden; 2 Department of Medical Radiation Sciences, Institute of Clinical Sciences, Sahlgrenska Academy, University of Gothenburg, Gothenburg, Sweden; 3 Rheumatology Clinic, Sahlgrenska University Hospital, Region Västra Götaland, Gothenburg, Sweden; 4 The First Affiliated Hospital, Guangzhou University of Chinese Medicine, Guangzhou, China; 5 Department of Ophthalmology, Sahlgrenska University Hospital, Region Västra Götaland, Gothenburg, Sweden; 6 Department of Clinical Immunology and Transfusion Medicine, Sahlgrenska University Hospital, Region Västra Götaland, Gothenburg, Sweden; 7 Division of Rheumatology, Department of Medicine Solna, Karolinska Institutet, Center for Molecular Medicine, Karolinska University Hospital, Stockholm, Sweden

**Keywords:** Arthritis, Rheumatoid, Hand Strength, Magnetic Resonance Imaging, Brain Mapping, Motor Cortex, CD4-Positive T-Lymphocytes, Monocytes, Cytokines, Gene Expression Profiling, Exercise Therapy.

## Abstract

**Background:**

Handgrip strength (HGS) in rheumatoid arthritis (RA) is commonly attributed to joint pathology, but may also reflect extra-articular manifestations, including atrophy of motor-related brain regions. We investigated HGS as a marker of peripheral joint status, systemic immune regulation, and central motor integrity.

**Methods:**

Maximal HGS was assessed using a dynamometer. Joint pathology was evaluated using radiographic and clinical measures, and upper limb disability using questionnaire. Brain volumes were quantified using MRI and MAPER software. Transcriptome sequencing was performed on circulating CD4⁺ and CD14⁺ cells. In a six-month, single-arm pilot trial, a subgroup of patients performed daily hand exercises. Associations and longitudinal changes were analysed using linear mixed-effects models accounting for repeated measurements across hands and timepoints, with variable selection performed using LASSO regression.

**Results:**

A total of 59 women with established RA were included in the cross-sectional analysis (median age 64 years [range 23–76], DAS28 2.46 [1.1–5.8], disease duration 11 years [0–45]). Lower HGS was associated with greater disability. HGS was also independently associated with premotor and supplementary motor cortex (PMA/SMA) volume after adjustment for age, hand dominance, and joint pathology. Among joint pathology measures, tender joint count showed a significant negative association with HGS. Transcriptome analyses of CD4⁺ and CD14⁺ cells indicated that lower HGS was associated with reduced immune responsiveness and altered cytokine signalling pathways. In a six-month pilot hand exercise trial (*n* = 12; median age 54 years [28–68], DAS28 2.87 [1.2–3.6], disease duration 14 years [1–40]), HGS increased at 3 months, with a non-significant trend at 6 months. Baseline PMA/SMA volume showed a non-significant trend towards predicting HGS improvement. Longitudinal analyses revealed region-specific brain changes, with a decrease in PMA/SMA volume and an increase in insular volume over time.

**Conclusions:**

Handgrip weakness in RA may reflect both joint pathology and motor cortex atrophy in the PMA/SMA. Hand exercise improved HGS and induced certain structural changes in the brain, though effects on motor regions remain uncertain and warrant further study.

**Trial registration:**

Clinical trial registration: ClinicalTrials.gov, NCT04378621. Registration date: May 5, 2020.

**Supplementary Information:**

The online version contains supplementary material available at 10.1186/s12916-026-04956-z.

## Background

Rheumatoid arthritis (RA) is a chronic inflammatory joint disease that frequently impairs hand function through pain, local inflammation, structural skeletal changes, and reduced strength [1]. Even in patients with low RA disease activity or clinical remission, handgrip strength (HGS) is often diminished early in the disease, affecting both average and peak force across age and sex groups [2]. HGS is widely used as a simple measure of physical function and overall health [3, 4] that typically peaks in early adulthood and declines from midlife onward [5]. In RA, HGS reflects hand disability and related variables, correlating with RA-associated joint damage and overall disease burden. However, the relationship between HGS and disease activity remains unclear, with conflicting results reported across studies [6, 7].

Recent neuroimaging studies suggest that HGS may also reflect changes in brain structure. In the general population, lower HGS has been linked to age-related alterations in white matter integrity [8], white matter hyperintensities [9], and decreased grey-matter volume in motor-related regions, including the basal ganglia (BG), thalamus (Tha), temporal lobe, and brainstem [10]. In neurodegenerative conditions, lower HGS was associated with reduced hippocampal and frontal lobe volume [11]. Reduced HGS has also been associated with chronic pain conditions, in chronic back pain reduced HGS was associated with lower grey-matter volume in sensorimotor regions [12]. Despite differing pathology, these findings indicate that HGS can relate to structural features of motor‑related brain regions in long‑standing conditions. This provides a rationale for examining whether similar associations may be present in RA. Research in both clinical and healthy populations indicates that targeted hand exercise can induce structural and functional changes in the brain, a phenomenon known as experience-dependent neuroplasticity (13, 14, 15]. For example, intensive HGS exercise in stroke patients improved motor recovery and promoted white matter plasticity, highlighting the potential for rehabilitative interventions to strengthen central motor networks [13]. Similarly, exercise novel hand-based motor skills can induce regionally specific structural changes. Juggling practice has been associated with increased grey-matter volume in the visual motion complex (hMT/V5) [14], and performance-related increases have been observed in dorsal parietal cortex and primary motor cortex after long-term juggling exercise [15].

**Fig. 1:**
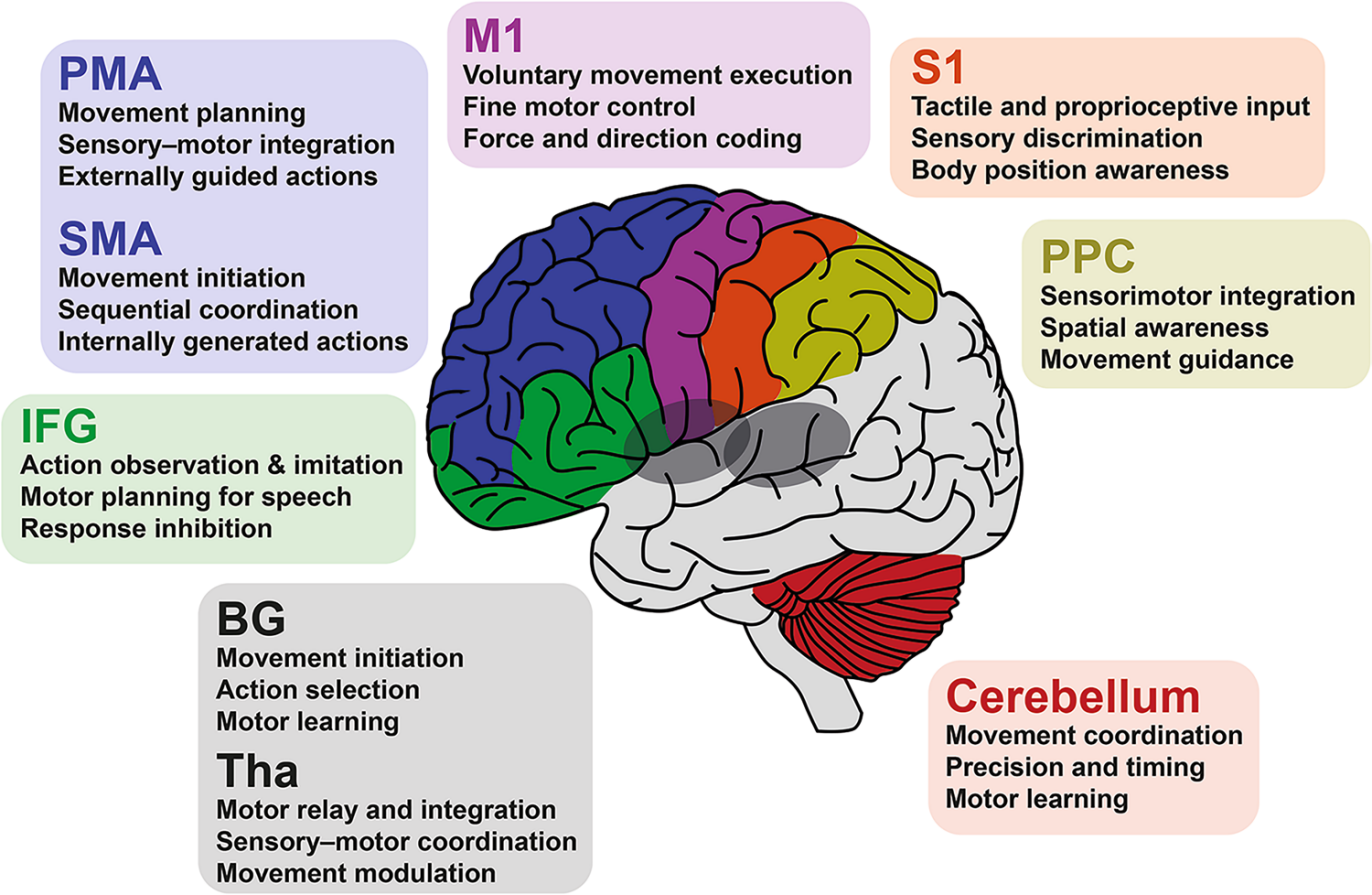
Location and main motor-related functions of motor brain regions. PMA, premotor area; SMA, supplementary motor area; M1, primary motor cortex; S1, primary somatosensory cortex; PPC, posterior parietal cortex; IFG, inferior frontal gyrus; BG, basal ganglia; Tha, thalamus.

HGS reflects not only peripheral joint function but also central motor control (Fig. 1). The primary motor cortex (M1) in the precentral gyrus initiates voluntary movement [16], while the primary somatosensory cortex (S1) in the postcentral gyrus processes sensory feedback and contributes to motor planning [17]. Premotor and supplementary motor areas (PMA/SMA), also part of the motor cortex and located in the middle and superior frontal gyri, guide complex movement sequences and coordination [18]. The inferior frontal gyrus (IFG), including pars opercularis, orbitalis, and triangularis, supports visuomotor integration [19]. The posterior parietal cortex (PPC), encompassing the superior parietal gyrus, supramarginal gyrus, and angular gyrus, integrates visuospatial information and may influence anticipatory control of the hand [20, 21]. Subcortical structures, including the BG (putamen, caudate, pallidum, subthalamic nucleus, substantia nigra) and thalamus, contribute to motor readiness and learning via cortico-subcortical loops [22, 23]. The cerebellum, spanning lobular and vermal regions, refines timing, coordination, and sensorimotor integration [24].

Previous research suggests that motor regions are affected in RA. Resting state functional Magnetic Resonance Imaging (MRI) revealed that RA patients had increased connectivity between pain seed regions in the SMA and the S1/M1, and between the insula and the PMA [25]. In the PreCePra phase 3 trial, higher baseline disease-associated central nervous system activation volume during compression of general tender joints predicted a greater likelihood of achieving low disease activity with tumour necrosis factor (TNF) inhibition (certolizumab pegol) in RA [26]. In experimental arthritis caused by overexpression of human TNF, mice developed inflammatory responses in frontal cortex, striatum and Tha [27]. Collagen-induced arthritis also led to increased inflammation in frontal cortex, striatum and hippocampus in rats [28] and mice [29].

Together, these findings suggest that both peripheral and central mechanisms contribute to impaired motor function in RA. We therefore investigated whether reduced HGS reflects not only peripheral joint pathology but also structural changes in motor-related brain regions, and whether targeted hand exercise is associated with changes in HGS and brain structure.

## Methods

### Design and study population

In this observational cross-sectional study with an embedded longitudinal intervention component, patients were enrolled from the Rheumatology Clinic at Sahlgrenska University between 2019 and 2020. Inclusion criteria were female patients over the age 18 with established RA fulfilling the 2010 American College of Rheumatology/European League Against Rheumatism (ACR/EULAR) classification criteria [30]. Exclusion criteria included contraindications to MRI (metallic foreign bodies including implantable medical devices; claustrophobia), severe physical or psychiatric illness, history of cerebrovascular disease, neurological disorders, any malignancy under treatment, previous brain surgery, ongoing antidepressant treatment, and inability to understand spoken and written Swedish. Patients were recruited for the study either through an invitation letter containing study information or by personal invitation during routine clinical visits. The study coordinator (STS) subsequently contacted interested patients by telephone to schedule a study visit. A subgroup consisting of patients on stable treatment with Janus kinase inhibitors (JAKi) were invited to participate in the non-randomized longitudinal interventional part of the study consisting of a six-month hand exercise program. At the baseline visit, all patients answered questionnaires and underwent clinical examination, blood sampling, assessment of hand function, and MRI. Patients enrolled in the hand exercise program were instructed to complete exercise diaries. They also attended follow-up hand function assessments at 3 and 6 months. At six months, the clinical examination, questionnaires, and MRI were repeated. All assessments were conducted as part of predefined study visits and were not part of routine clinical care. See full timeline in Table 1.

**Table 1:** Study overview

Assessment	Baseline (*n* = 59)	3 months (*n* = 12)	6 months (*n* = 12)
Hand exercise programme	instructed	ongoing	completed
Handgrip strength	✓	✓	✓
Study diary	✓	✓	✓
Clinical examination	✓	not done	✓
Blood sampling	✓	not done	not done
Questionnaires	✓	not done	✓
MRI of the brain	✓	not done	✓
Radiographs of hands	✓	not done	not done

A check mark (✓) indicates that the assessment or procedure was performed at the corresponding visit. MRI, magnetic resonance imaging.

### Clinical and anthropometric assessment

Age, disease duration (DD, years since diagnosed) and medication use were obtained from medical records. Patients underwent assessment of swollen joint count (SJC) and tender joint count (TJC) using the standard 28-joint count [30]. In addition to the total SJC and TJC, hand-specific SJC and TJC were recorded separately for each hand (dominant and non-dominant, left and right) to capture side-specific joint involvement. Anthropometric measurements recorded included body weight and height for calculation of body mass index (BMI) and systolic blood pressure (SBP). Blood samples were collected and sent to the Clinical Chemistry Laboratory, Sahlgrenska University Hospital, for analysis of systemic inflammatory markers, including C-reactive protein (CRP) and erythrocyte sedimentation rate (ESR). ESR was used to calculate the disease activity score, 28-joint count (DAS28) with three variables [31]. All patients completed validated Swedish self-report questionnaires: the Disabilities of the Arm, Shoulder and Hand (DASH) [32], Health Assessment Questionnaire (HAQ) [33], and the Fibromyalgia Impact Questionnaire (FIQ) [34].

### Assessment of joint skeletal damage

Posterior-anterior radiographs of both hands were obtained to evaluate bone erosions and joint-space narrowing. These structural changes were quantified using the Sharp scoring method as modified by van der Heijde (SHS) [35], which yields a composite score reflecting both erosive and cartilage pathology. For both hands, the total SHS range 0-280. The range for erosion score (ES) is 0–80, and the range for joint space narrowing (JSN) is 0–60 in each hand.

### Handgrip assessments

HGS was assessed using the Grippit device (AB Detektor, Gothenburg, Sweden), as described in an established and validated protocol [36, 37], by an experienced occupational therapist. Both hands were tested in turn, with three consecutive trials performed for each hand. For each trial, grip force was recorded as maximum, mean, and sustained force (Newton, N). For each parameter, the mean across the three trials was calculated. For the analyses, the maximum grip force was used to represent HGS and focused on the dominant and non-dominant hand. In parallel, thumb opening size (TOS) was measured to evaluate hand positioning. Self-reported handedness was recorded.

### Hand exercise trial

A subgroup of patients on stable treatment with JAKi participated in a structured six-month hand exercise intervention study. The exercise program consisted of five standardised movements targeting fingers, wrist, and thumb, adapted from a validated protocol [38]. Exercises included flexion/extension, radial/ulnar deviation, and thumb abduction/opposition, performed daily for five minutes per hand. An experienced occupational therapist provided instruction at the program’s initiation and hand function assessment at baseline and at the 3- and 6-month follow-up. Patients completed predefined exercise diaries, which they returned at 3- and 6-months. The diary was structured on exercise adherence (yes/no), medication use including analgesics (yes/no), and physical activity (yes/no). Hand pain and fatigue were rated on visual analogue scales ranging from 0 for “no pain/fatigue” to 100 for “worst imaginable pain/fatigue”. General well-being was recorded using a five-level mood scale represented by smiley icons. In an additional free-text field, patients described the type of physical activity and number of steps or provided other comments.

### MRI of the brain

High-resolution MRI was performed on a 3-Tesla Philips Gyroscan Achieva scanner equipped with a 32-channel SENSE head coil (Philips Healthcare). Cranial T1-weighted images were acquired with the following parameters: flip angle = 8°, echo time (TE) = 4.0 ms, repetition time (TR) = 8.4 ms, SENSE factor = 2.7, turbo field echo factor = 240, and isotropic voxel size = 1 ⋅ 1 ⋅ 1 mm.

Pre-processing included correction for field inhomogeneities using N4ITK. Morphometric analysis was performed on the T1-weighted images using ensemble machine learning models for semantic segmentation. Brain extraction (skull stripping) was conducted with Pincram [39], and anatomical labelling was performed using MAPER [40]. The MAPER pipeline utilised exercise data from 30 healthy volunteers included in the Hammers Adult Brain Atlas Database (41, 42, 43, 44], with expert-defined reference labels covering 120 cortical and subcortical regions (pre-release version).

Tissue classification was based on three-class probability mapping (seg_EM from NiftySeg, https://github.com/KCL-BMEIS/NiftySeg), distinguishing grey matter, white matter, and cerebrospinal fluid. Crisp label images were derived from these probability maps. For each bilateral region, left and right volumes, grey matter, white matter and cerebrospinal fluid volumes were summed to obtain total regional volumes. All volumetric measures were normalised by intracranial volume as determined by Pincram.

To specifically investigate motor-related brain volume, regions were defined based on the Hammers Adult Brain Atlas Database and included in the analysis as functionally grouped motor regions. These composite regions were constructed by combining anatomically defined subregions. For example, PMA/SMA was derived from portions of the superior and middle frontal gyri. A full list of included regions and their functional annotations is provided in Table 2.

**Table 2:** Motor-related brain regions

Motor region [45]	Abbreviation	Sub-areas	Function
Primary motor cortex	M1	Precentral gyrus	Voluntary motor control
Primary somatosensory cortex	S1	Postcentral gyrus	Sensory feedback to guide motor control
Premotor area and supplementary motor area	PMA/SMA	Middle frontal gyrus Superior frontal gyrus	Motor planning, coordination, internally generated movement
Inferior frontal gyrus	IFG	Inferior frontal gyrus pars opercularis/orbitalis/triangularis	Motor inhibition, language-motor integration
Posterior parietal cortex	PPC	Superior parietal gyrus Supramarginal gyrus Angular gyrus	Spatial coordination, sensorimotor feedback
Basal ganglia and Thalamus	BG/Tha	Putamen Caudate nucleus Palladium Subthalamic nucleus Substantia nigra Thalamus	Basal ganglia-thalamocortical motor loop, regulates motor execution
Cerebellum	Cerebellum	Anterior lobe Superior posterior lobe Inferior posterior lobe Flocculonodular lobe Corpus medullare Vermis	Fine motor control, motor learning, coordination

### Cell isolation and sorting

Peripheral blood samples were collected between 07:00 and 10:00 AM. Venous blood was drawn from the cubital vein into vacuum tubes (BD Vacutainer) for serum preparation. Serum samples and culture supernatants were stored at − 70 °C until analysis.

Peripheral blood mononuclear cells (PBMCs) were isolated from heparinised blood by density gradient centrifugation using Lymphoprep (Axis-Shield PoC As, Norway). Cluster of Differentiation (CD)4⁺ and CD14⁺ cells were enriched from the PBMC fraction via positive selection (CD4: Invitrogen, Waltham, MA, USA, 11331D; CD14: StemCell Technologies, Vancouver, Canada, 17858). Flow cytometric analysis confirmed a sorted cell purity ranging from 78% to 90%.

Separate CD4⁺ and CD14⁺ cell cultures were established by seeding cells at a density of 1.25 × 10⁶ cells/mL in RPMI 1640 medium supplemented with 5% foetal bovine serum (Sigma-Aldrich), 2 mM Glutamax (Gibco, Waltham, MA, USA), 50 µg/mL gentamicin (Sanofi-Aventis, Paris, France), and 50 µM β-mercaptoethanol (Gibco). CD4⁺ cells were stimulated for 2 h on culture plates coated with anti-CD3 monoclonal antibodies (0.5 µg/mL; OKT3, Sigma-Aldrich, St. Louis, MO, USA). CD14⁺ cells were stimulated with 5 µg/ml lipopolysaccharide (LPS, Sigma-Aldrich). Cultures were maintained at 37 °C in a humidified incubator with 5% CO₂. Supernatants were harvested for downstream analysis of secreted cell products (see below). Cells were used for RNA sequencing.

### RNA sequencing and analysis

RNA was extracted from CD4⁺ [46] and CD14⁺ [47] cell cultures using the Total RNA Purification Micro Kit (Norgen Biotek Corp, Thorold, Ontario, Canada). RNA quality was assessed with an RNA 6000 Pico Kit on the 2100 Bioanalyzer System (Agilent Technologies, Inc., Santa Clara, CA, USA). RNA sequencing was performed using a Hiseq 2000 System (Illumina, Inc., San Diego, CA, USA) at the BEA Core Facility, Karolinska Institutet, Sweden.

Transcript mapping to the genome was conducted using the UCSC Genome Browser annotation for the hg38 human genome assembly. The transcriptome data were filtered to retain genes with mean normalised count ≥ 5 and normalised by size factors using the DESeq2 package [48] (version 1.40.2) in R. Differential expression was analysed with the DESeq2 Wald test, with Benjamini-Hochberg adjustment of p-values.

Variance-stabilised counts were batch-corrected with the limma package [49] (version 3.56.2). Gene set enrichment analysis of KEGG pathways was performed using the clusterProfiler package [50] in R based on ranked log2 fold changes of all genes.

### Enzyme-linked immunosorbent assay (ELISA)

Cytokine levels were measured in cell culture supernatants from aCD3-stimulated CD4^+^ and from LPS-stimulated CD14⁺ cells from RA patients, using sandwich ELISAs for TNF (M1923) and IFNγ (M1933) (Sanquin, Amsterdam, The Netherlands); IL1β (DY201) and IL10 (DY217B) (R&D Systems, Minneapolis, MN, USA).

### Ethical consideration

All patients gave written informed consent prior to participating in the study. The study was approved by the Swedish Ethical Review Authority (Dnr. 2019–03787) and was conducted in accordance with the Declaration of Helsinki. The trial is registered at ClinicalTrials.gov (ID NCT04378621).

### Statistical analyses

Analyses were designed to evaluate associations between HGS and motor-related brain regions, and to assess longitudinal changes following hand exercise.

All statistical analyses were performed in R [51] version 4.3.1 (R Foundation for Statistical Computing) or GraphPad Prism 10 version 10.5.0 running on macOS. Linear mixed-effects models (LMM) were fitted using the R packages lme4 (v1.1-37) and lmerTest (v3.1-3).

Functional disability was assessed using the DASH questionnaire. Each DASH subscale (hand, arm, and shoulder) was analysed with separate LMMs, with HGS as the dependent variable and the corresponding DASH score as a fixed effect. Hand dominance was included as a covariate, and patient ID was modelled as a random intercept to account for repeated measurements across hands.

Associations between HGS and motor-related brain regions were analysed using LMM with HGS as the dependent variable. Volumes of predefined motor-related brain regions and clinical variables were included as candidate predictors. Clinical variables were selected based on clinical relevance for hand function, and included age, DD, hand dominance, CRP, SJC, TJC, SHS, and TOS, with hand-specific values of SJC, TJC, SHS, and TOS used in the analyses. Given the large number of candidate predictors relative to the sample size, LASSO (Least absolute shrinkage and selection operator) regression was used for variable selection to identify the variables most strongly associated with HGS while reducing the risk of overfitting. Hand dominance and age were forced into the model. Predictors retained by LASSO were then entered into the main LMM, with selected brain region volumes and covariates included as fixed effects, and patient ID was included as random intercept. Before modelling, all continuous variables were screened for distributional skewness. Variables with ∣“skewness”∣ >1 were log transformed to reduce right skew and improve model fit, while variables with lower skewness were retained on their original scale. Missing numeric values were imputed using the median, and continuous predictors were standardised (z-scores). The extent of missing data for each variable is reported in Additional file 1: Table S1. Model comparison was performed using the corrected Akaike Information Criterion (AICc), which was used to evaluate relative model fit and guide selection between competing models.

Additionally, associations between HGS and general health were analysed using LMM. HGS was included as the dependent variable, and HAQ score, FIQ total score, BMI, and SBP were included as fixed effects. Hand dominance was included as a covariate, and patient ID was included as random intercept. Continuous variables were log-transformed when skewed and subsequently standardised (z-scores). Missing values were limited (< 5% across variables) and were handled using median imputation. Model estimates are reported as fixed effects with 95% confidence intervals.

To investigate the associations between gene expression and clinical or imaging variables, two different design formulas were applied in the RNA-seq analyses. The first formula included HGS, age, and batch; and the second included SHS, age, and batch, where HGS, PMA/SMA volume, SHS, and age were treated as continuous variables. In all analyses, “batch” was included in the design formula because the samples were sequenced in two separate batches.

A subset of patients participated in two follow-up visits after 3 and 6 months, where hand strength (3 and 6 months) and magnetic resonance volumetry (6 months) were repeated after performing daily hand exercises. LMM was used to evaluate HGS over time, with time after exercise (baseline, 3 months, and 6 months) as a fixed effect, dominant hand and adherence to the program as covariates, and patient ID as random intercept. To evaluate whether baseline brain structure predicted exercise response, separate mixed-effects models were fitted with HGS as the outcome, time and standardised brain region volume as predictors, dominant hand as a covariate, and a random intercept for patient. To examine longitudinal brain structural changes during the intervention, mixed-effects models were fitted with standardised brain region volume as the outcome and time as predictor, and with patient as a random intercept. Changes in HGS between baseline and 6 months were analysed using models with ΔHGS as the outcome and standardised changes in brain region volume (ΔInsula) and dominant hand as predictors.

## Results

### Study population and HGS characteristics

A total of 60 female patients with RA were recruited from the Rheumatology Clinic at Sahlgrenska University Hospital. One patient was excluded due to incomplete MRI data, leaving 59 patients included in the cross-sectional analyses (Additional file 2: Fig. S1). The cohort comprised 47 patients without exercise and 12 patients included in the hand exercise trial. The median age was 64 years [interquartile range 52–68], median DAS28 was 2.46 [1.75–3.17], and median DD was 11 years [6–19]. HSG was assessed in all patients. Median dominant HGS was 176 N [123–248], and median non-dominant HGS was 188 N [122–241] (Table 3). A strong correlation was observed between dominant and non-dominant HGS (Spearman’s *r* = 0.95, *p* < 0.0001, 95% confidence interval: 0.93–0.97).

**Table 3:** Clinical and demographic variables of the study patients

	Variable	All patients (*n* = 59)	Non-exercise group (*n* = 47)	Hand exercise group (*n* = 12)
Demographics	Age, years	64 [52–68]	66 [56–69]	54 [46–64]
	DD, years	11 [6–19]	10 [6–19]	14 [9–20]
	BMI, kg/m²	25.6 [23–28]	26.7 [23–28]	24.5 [22–26]
	SBP, mmHg	135 [123–144]	137 [123–144]	134 [116–138]
Disease activity	DAS28 (AU)	2.46 [1.8–3.2]	2.39 [1.7–3.2]	2.87 [2.6–3.2]
	Swollen joints, n	1 [0–2]	0 [0–1.5]	2 [1.8–3.25]
	Tender joints, n	1 [0–5]	0 [0–2]	4.5 [3.8–5.5]
Questionnaires	DASH total score	42.5 [22–57]	42.5 [22–57]	39.3 [17–52]
	HAQ total score	0.38 [0–1]	0.37 [0.1–1]	0.6 [0–0.7]
	FIQ total score	27.5 [16–38]	27.5 [16–38]	28.5 [14–39]
Inflammation	CRP, mg/mL	1 [1–2.75]	1 [1–3]	1 [1–1.5]
Joint damage	Sharp total score	27 [11.7–53.7]	27 [12.8–53.8]	29 [9.3–50.5]
Handgrip strength	Dominant	176 [123–248]	176 [124–248]	166 [151–203]
	Non-dominant	188 [122–241]	188 [124–248]	184 [151–203]
Dominance	Right-handed, %	97% (57/59)	98% (46/47)	92% (11/12)

Values are presented as median [interquartile range, 25–75] or percentage (n/N). Abbreviations: Disease duration; BMI, body mass index; SBP, systolic blood pressure; DAS28, Disease Activity Score based on 28 joints; DASH, Disabilities of the Arm, Shoulder and Hand questionnaire; HAQ, Health Assessment Questionnaire; FIQ, Fibromyalgia Impact Questionnaire; CRP, C‑reactive protein.

### Reduced HGS was associated with functional disability

**Fig. 2:**
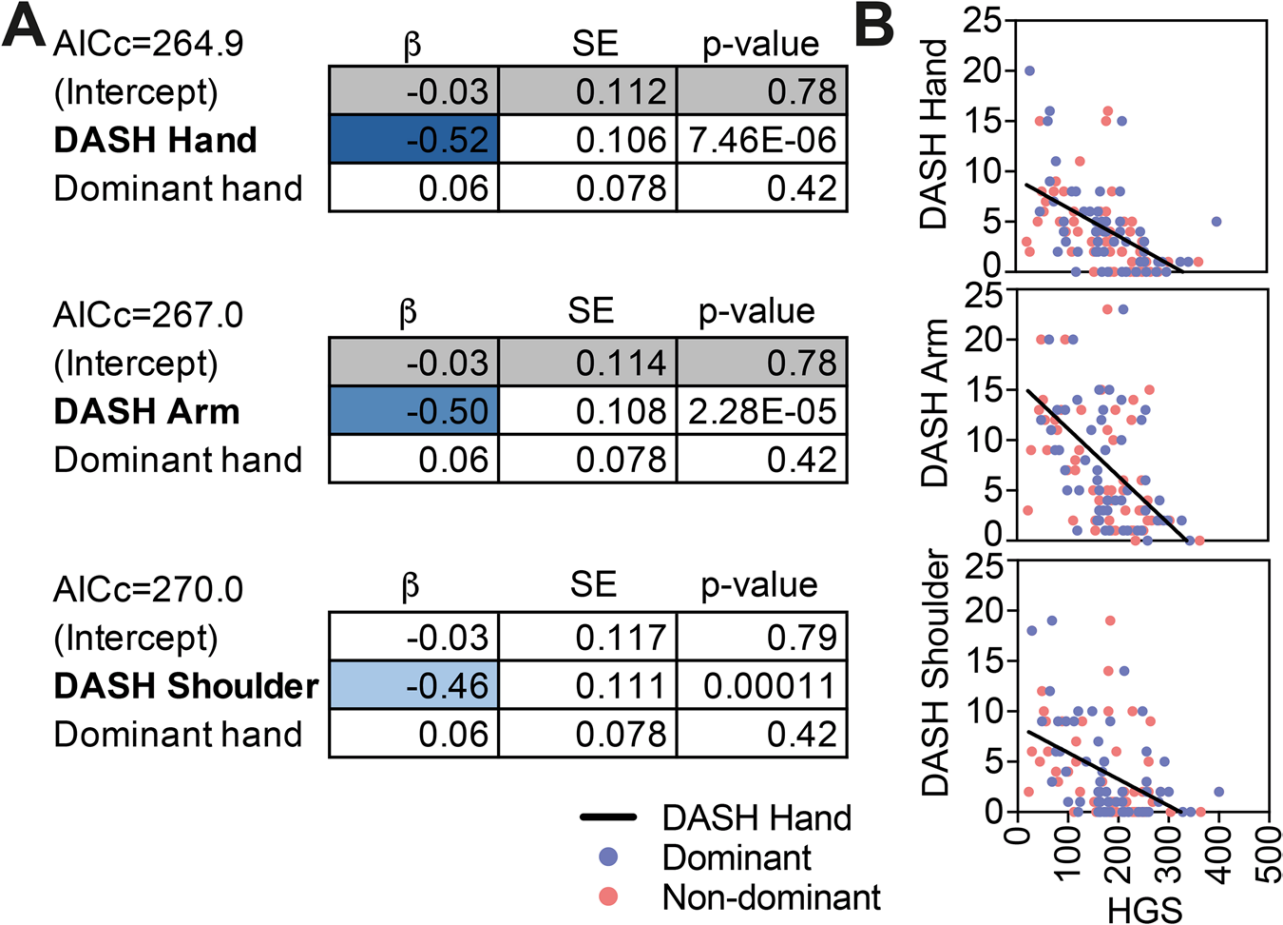
Reduced handgrip strength (HGS) was associated with functional disability**. (A)** Associations between HGS and self-reported upper-limb function were examined using linear mixed-effects models with DASH subscales (Disabilities of the Arm, Shoulder and Hand) as outcomes and HGS as the main predictor, adjusting for hand dominance and including a random intercept for patient ID. β, beta estimate; SE, standard error; AICc, corrected Akaike Information Criterion. **(B)** Scatterplots illustrating the relationship between HGS and each DASH subscale. Data points are colour-coded by hand dominance, and each plot includes a fitted regression line

HGS was significantly associated with functional disability. In LMMs including patient ID as a random intercept and hand dominance as a covariate, higher DASH scores were consistently linked to lower HGS (Fig. 2A). For the DASH Hand model, greater disability was associated with reduced HGS (β = − 0.52, SE = 0.1, df = 57, t = − 4.93, *p* = 7.46 × 10⁻⁶; AICc = 264.9). A similar pattern was observed for DASH Arm (β = − 0.50, SE = 0.108, df = 57, t = − 4.62, *p* = 2.28 × 10⁻⁵; AICc = 267.0) and DASH Shoulder (β = − 0.46, SE = 0.111, df = 57, t = − 4.16, *p* = 1.1 × 10⁻⁴; AICc = 270.0). The fixed effect of dominance was not significant in any model (β = 0.06, SE = 0.08, df = 58, t = 0.81, *p* = 0.42), indicating that the association between reduced grip strength and greater upper-limb disability was similar in dominant and non-dominant hands. The corresponding scatterplots (Fig. 2B) illustrate the same pattern visually, with lower HGS values clustering alongside higher DASH scores across all three subscales, and with no clear separation between dominant and non-dominant hands. In addition to DASH, HGS was associated with functional status as measured by the HAQ score (Additional file 3: Table S2), whereas no significant associations were observed with general health indicators such as BMI or SBP.

### Motor-related brain regions and joint pathology were associated with reduced HGS

**Fig. 3:**
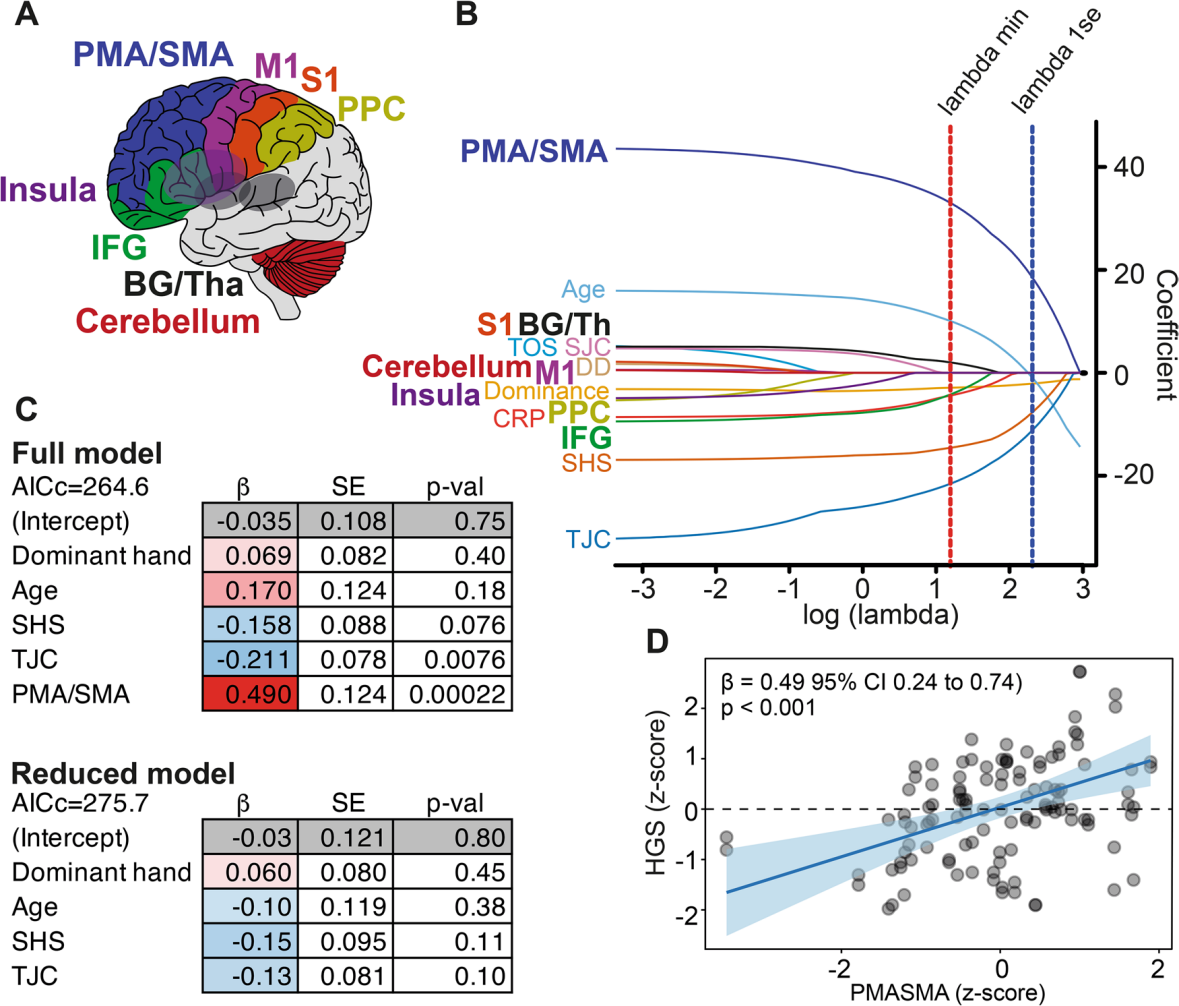
Motor-related brain regions and joint pathology were associated with reduced handgrip strength (HGS). **A**) Motor-related brain regions included the primary motor (M1) and somatosensory (S1) cortices, premotor and supplementary motor areas (PMA/SMA), inferior frontal gyrus (IFG), posterior parietal cortex (PPC), basal ganglia and thalamus (BG/Tha), and cerebellum. **B**) Least absolute shrinkage and selection operator (LASSO) coefficient paths for all motor-related regions together with hand-specific pathology measures: Sharp/van der Heijde Score (SHS), tender joint count (TJC), swollen joint count (SJC), and thumb opening size (TOS). Additional predictors included C-reactive protein (CRP), disease duration (DD), age, and hand dominance. Vertical lines indicate lambda min and lambda 1SE. **C**) Results of a linear mixed-effects models (LMM). The full model included PMA/SMA volume, age, SHS, TJC, and hand dominance with participant ID as a random intercept, whereas the reduced model included age, SHS, TJC, dominance, and participant ID as a random intercept. β, beta estimate; SE, standard error; AICc, corrected Akaike Information Criterion. **D**) Scatterplot illustrating the association between PMA/SMA volume and HGS, with the fitted regression line derived from the LMM. All variables were z-scored prior to modelling.

We next examined where this functional decline relates to brain volume in regions involved in movement planning and execution while accounting for joint pathology (Fig. 3A). Using the lambda 1SE criterion, the LASSO procedure selected PMA/SMA volume along with SHS and TJC as the most informative predictors (Fig. 3B). These variables were then entered into a LMM with HGS as the dependent variable, PMA/SMA as the primary predictor, SHS and TJC as covariates, dominance and age included as a fixed effect, and patient ID as a random intercept. In this model HGS was significantly associated with PMA/SMA volume (β = 0.49, SE = 0.12, t = 3.96, *p* < 0.001), indicating that individuals with larger PMA/SMA volume exhibited higher grip strength (Fig. 3C). TJC also showed a significant negative association with HGS (β = − 0.21, SE = 0.08, t = − 2.71, *p* = 0.008), while SHS showed a negative but non-significant trend (β = − 0.16, SE = 0.09, t = − 1.79, *p* = 0.076). Hand dominance did not contribute significantly (β = 0.07, SE = 0.08, t = 0.80, *p* = 0.40). Age was also not significantly associated with HGS (β = 0.17, SE = 0.12, t = 1.37, *p* = 0.18). Adding PMA/SMA volume to a model containing only age, SHS, TJC, dominance and ID significantly improved model fit (AICc = 264.6 vs. 275.7; likelihood-ratio *p* < 0.001). In summary, PMA/SMA volume was significantly associated with HGS (Fig. 3C–D).

### Handgrip weakness was associated with reduced responsiveness of circulating CD4⁺ and CD14⁺ cells

To further understand contributing factors to HGS loss in RA, we analysed the transcriptome of CD4⁺ and CD14⁺ cells sorted from peripheral blood of the patients at the day of brain imaging, while correcting for age.

**Fig. 4:**
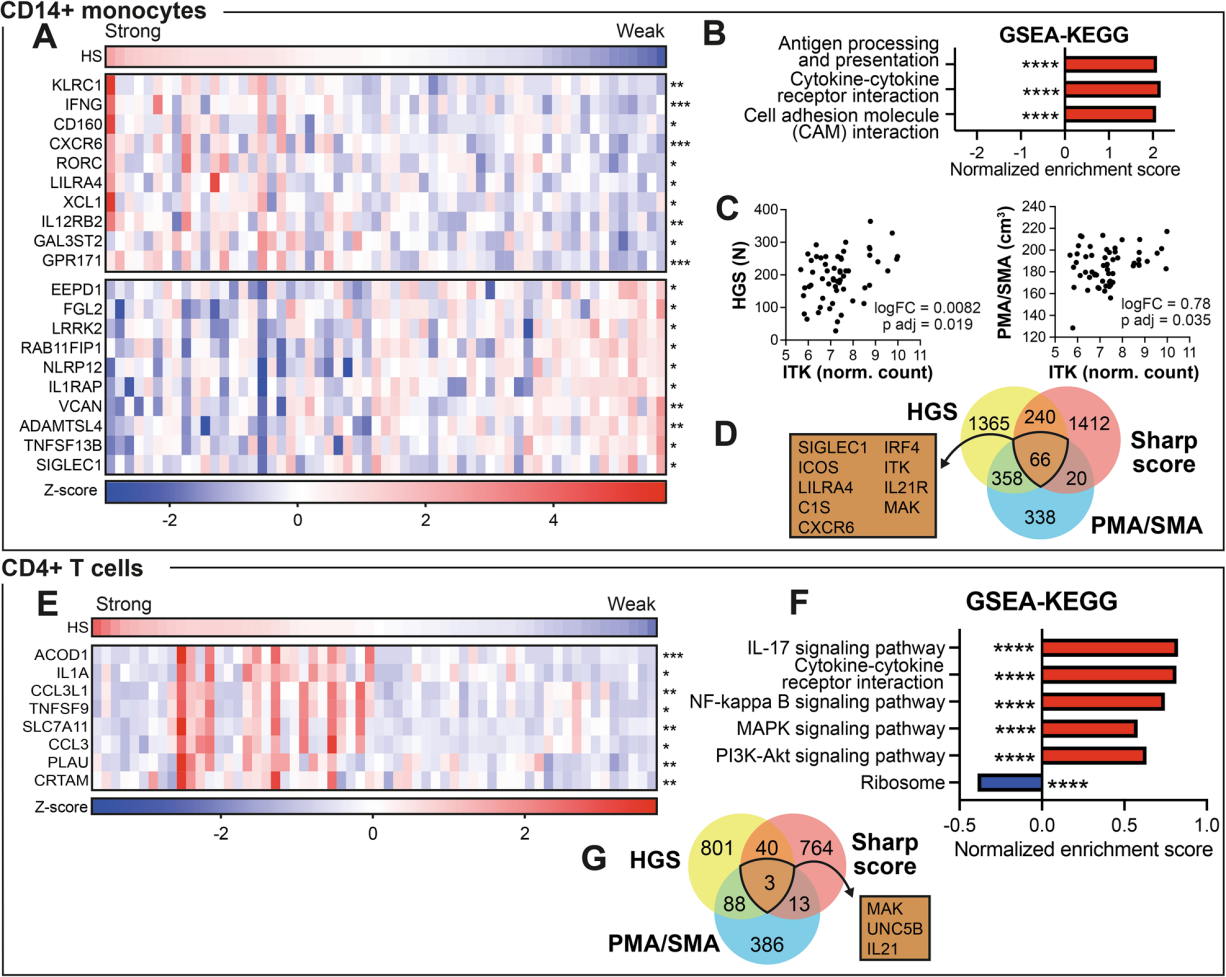
Handgrip weakness was associated with reduced responsiveness of circulating CD4⁺ and CD14⁺ cells. **A**) Normalised expression of genes associated with strong (top 10 genes) and weak (top 10 genes) hands in CD14⁺ cells, according to Wald’s test using handgrip strength (HGS) as a continuous variable and controlling for age and batch. **B**) GSEA-KEGG pathway analysis of genes from CD14⁺ cells. **C**) Expression of ITK (normalised counts) plotted against HGS (left) and the volume of the premotor area/supplementary motor area (PMA/SMA, right). Log2 fold change (log2 FC) and the adjusted p-value (p adj) was calculated with Wald’s test, while controlling for age and batch. **D**) Venn diagram of the number of genes with a p-value under 0.05 when the analysis is run with HGS, PMA/SMA volume or Sharp score of the hands as the independent variable. A selection of the overlapping genes is presented in the brown box. **E**) Normalised expression of genes associated with strong hands in CD4⁺ cells, according to Wald’s test using HGS as a continuous variable and controlling for age and batch. All significant genes are included. **F**) GSEA-KEGG pathway analysis of genes from CD4⁺ cells. **G**) Venn diagram of the number of genes with a p-value under 0.05 when the analysis is run with HGS, PMA/SMA volume or Sharp score as the independent variable. The overlapping genes are presented in the brown box. Statistical significance is marked by asterisks (*p < 0.05, **p < 0.01, ***p < 0.001, ****p < 0.0001).

In CD14⁺ cells, we found 115 genes that were significantly associated with the HGS of the strongest hand (adjusted p-value < 0.05, Wald’s test using continuous variables). The 10 top genes upregulated and downregulated with increasing HGS (by log2FC) are presented in Fig. 4A. Gene set enrichment analysis of KEGG pathways revealed a positive association between higher HGS and cytokine-cytokine receptor interaction, antigen processing and presentation and cell adhesion molecule interaction (Fig. 4B). Only one gene, the Interleukin (IL)-2-Inducible T-cell kinase (ITK, logFC = 0.000047, p_adjusted_ = 0.035), was associated with PMA/SMA volume after correcting for age. This gene had also a positive association to HGS (logFC = 0.0082, p_adjusted_ = 0.014, Fig. 4C). Additionally, we identified the genes of CD14⁺ cell transcriptome associated with the total SHS of hands and with PMA/SMA volume as the independent continuous variables. We found 66 common genes associated with HGS, SHS and PMA/SMA volumes, including SIGLEC1, ICOS, LILRA4, C1S, CXCR6, IRF4, ITK, IL-21R and MAK (Fig. 4D) characteristic for IFN-primed monocytes forming immune communication nodes.

In CD4⁺ T cells, only 8 genes were significantly associated with stronger HGS (Fig. 4E), including chemokine ligands CCL3 and CCL3L1, and cytokine IL-1 A. Gene set enrichment analysis of KEGG pathways revealed an association between HGS and cytokine-cytokine receptor interaction, IL-17 signalling, NF-kappa B signalling, PI3K/AKT signalling and MAPK signalling and an association between weaker HGS and the ribosome pathway (Fig. 4F). Intersecting the gene sets linked to HGS, SHS, and PMA/SMA volume yielded three candidates: MAK, UNC5B (netrin receptor UNC5B), and IL-21 (Fig. 4G). MAK and UNC5B are best known for roles in neuronal axon guidance and vascular patterning, whereas IL-21 is induced downstream of the ICOS–STAT3–BATF–IRF4 pathway and promotes sustained T cell help and effector responses in chronic immune activation.

### Hand exercise was associated with changes in HGS and brain structure

**Fig. 5:**
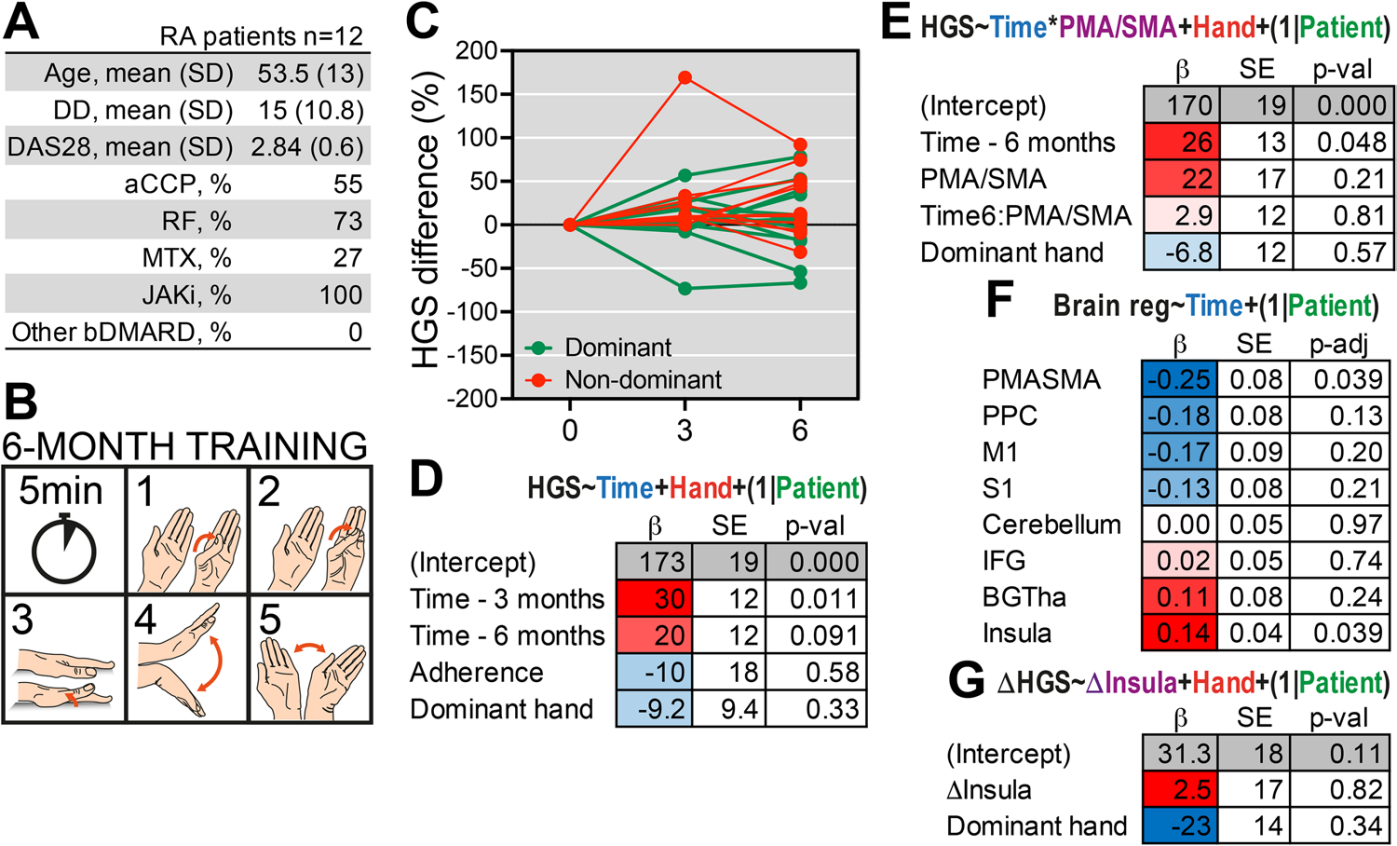
Hand exercise was associated with changes in handgrip strength (HGS) and brain structure. **A**) Characteristics of the 12 rheumatoid arthritis (RA) patients included in the hand exercise pilot study. **B**) Illustration of the five daily hand exercises performed during the 6-month exercise program. **C**) Percent change in HGS over time in the dominant and non-dominant hand. **D**) Results of a linear mixed-effects model (LMM) analysis. Dominant versus non-dominant hand was included as a covariate and patient ID as a random effect. **E**) LMM analysis of the association between training (time) and HGS when controlling for baseline premotor/supplementary motor area (PMA/SMA) volume. **F**) LMM analysis of the longitudinal changes in brain region volumes. The models (one per brain region) including time, HGS, and dominant hand as fixed effects and patient ID as a random intercept. P-values were adjusted for multiple hypotheses with the Benjamini-Hochberg (BH) method. β, beta estimate, SE, standard error. **G**) LMM analysis of the difference in HSG (ΔHSG) and difference in Insula volume (ΔInsula), dominant hand as fixed effects and patient ID as a random intercept.

In a pilot trial, 12 patients (Fig. 5A) completed 6 months of daily hand exercise (Fig. 5B), followed by repeat HGS assessment and brain MRI. Adherence was high, with a median of 85% (range 73–100%) based on daily diaries.

The patients included in the trial had a median age of 54 years [interquartile range 46–64], DD of 14 years [9–20] and DAS28 2.9 [2.6–3.2] (Fig. 5A; Table 3). They were all treated with JAKi for a duration of 1.2 years on average (1.7 months to 3.2 years before baseline), and 3 patients were also treated with methotrexate. One of the twelve patient was left-handed. The change in HGS of the dominant and non-dominant hand is illustrated in Fig. 5C. HGS increased significantly at 3 months (β = 30.3, SE = 11.5, t = 2.63, *p* = 0.011, Fig. 5D) and showed a non-significant trend at 6 months (β = 19.8, SE = 11.5, t = 1.72, *p* = 0.091), indicating early functional improvement. Dominant versus non-dominant hand had no significant effect (*p* = 0.33). We also evaluated whether baseline PMA/SMA volume influenced exercise-induced HGS changes. There was a non-significant trend for larger baseline PMA/SMA predicting greater HGS improvement (β = 22.3, SE = 16.9, *p* = 0.21, Fig. 5E). Accounting for baseline PMA/SMA, 6-month HGS was significantly higher (β = 25.9, SE = 12.6, t = 2.05, *p* = 0.048).

We next examined whether hand exercise affected brain regions associated with motor function. PMA/SMA volume decreased over 6 months (β = -0.25, SE = 0.080, t = -3.11, adjusted *p* = 0.039, Fig. 5F), indicating that measurable atrophy occurs in patients with RA despite hand exercise. In contrast, there was an increase in the insula (β = 0.14, SE = 0.043, t = 3.21, adjusted *p* = 0.039). Other motor-related regions (M1, S1, cerebellum, PPC, IFG, BG/Tha) did not change significantly (all *p* > 0.05). To assess whether increase in insula volume was linked to improvements in HGS, we tested associations between differences in HGS and differences in brain region volume. An increase in the insula was not correlated to changes in HGS (Fig. 5G).

## Discussion

This study demonstrated that reduced HGS in RA is associated with smaller volumes in the PMA/SMA, regions in the frontal lobe that guide complex movement sequences and coordination [17]. In the final model, PMA/SMA volume remained a significant predictor of HGS after adjustment for clinical covariates, with better model fit compared to a model excluding this region. These findings suggest that motor impairment reflects not only peripheral joint pathology but also structural changes in motor-related brain networks. Although M1 is traditionally considered the principal cortical region responsible for voluntary hand movements [51], PMA/SMA emerged as the strongest correlate of HGS. This is consistent with evidence that these regions are particularly engaged during resistance movements [52]. Among clinical variables, TJC was also significantly associated with reduced HGS, supporting a role of pain-related factors in limiting motor performance. In contrast, joint structural damage quantified by the SHS showed only a non-significant trend, suggesting that structural joint damage alone may not fully explain reduced grip strength in this cohort. In RA, the reduced volume in PMA/SMA could influence the efficiency of motor commands to the hand, contributing to diminished HGS. Functional imaging studies further support altered SMA and S1 connectivity in RA compared to healthy controls [25] reinforcing the involvement of motor network alterations in the disease.

Considering RA’s impact on motor-related brain regions, we examined whether targeted hand exercises could improve HGS and counteract the identified structural deficit. The exercise program is used clinically to improve joint flexibility and mobility and general function of the hands of RA patients. In our study, daily hand exercises in RA patients led to improved HGS after 3 months of exercise, indicating early functional responsiveness. At 6 months, we observed a trend to an improvement, and when we included the PMA/SMA volume in the model, it could also predict HGS at 6 months. This observation is consistent with earlier findings showing that regular hand exercise can improve hand function and HGS in RA [53]. However, given the small sample size, these observations should be considered exploratory and descriptive.

Morphometric brain assessment revealed that the volume of the PMA/SMA declined after 6 months of exercise. While this change cannot be directly attributed to the intervention, it may reflect ongoing disease-related or age-related processes previously described in the literature [54, 55]. However, given that hand function was likely influenced by the exercise intervention, this finding does not rule out a relationship between structural decline and function, but rather suggests that functional performance may have been maintained through other mechanisms. The insula, which is located adjacent to the PMA/SMA, increased in volume during the intervention period. However, we did not observe an association between changes in insular volume and changes in HGS, indicating that insular structural changes may not be directly linked to functional improvement in this cohort. Previous studies have reported associations between insular volume and HGS in the general population [10]. The insula is involved in processing perceived pain and effort [56], which may be relevant in RA, where joint pain can influence movement. However, our findings do not support such a relationship. Given the small sample size, these results should be interpreted with caution, and further studies are warranted to clarify the role of the insula and other motor-related brain regions in supporting HGS in RA during hand interventions. In conclusion, hand exercises may help preserve hand function in RA despite concurrent decline in the PMA/SMA volume. Although insular volume increased, its relationship with HGS remains unclear.

Transcriptomic analyses of circulating CD14⁺ and CD4⁺ cells suggested that reduced HGS in RA is associated with a less responsive immune profile. In both cell types, gene set enrichment analysis indicated enrichment of cytokine-related signalling pathways among genes positively associated with HGS, while weaker HGS was linked to reduced representation of these pathways. At the gene level, overlap analyses identified a small number of shared candidates, including IL-21 and IL-21R, which are involved in joint pathology and immune activation [57, 58]. Together with the association between HGS and PMA/SMA volume, these findings suggest that reduced grip strength may reflect broader alterations in systemic immune activity alongside central and peripheral disease processes. Further studies are needed to clarify the mechanisms underlying these associations.

We would like to point out several limitations of this study. First, our study was not designed to establish a causal relationship between motor brain region atrophy and hand weakness. While we were able to demonstrate an association between PMA/SMA volume and HGS, further studies are needed to investigate whether reduced HGS and local joint pathology cause motor brain region atrophy through inactivity or pain, or whether motor-region atrophy is primarily caused by an inflammatory mechanism. Second, our study showed only a weak association between HGS and the RA disease activity; our results align with some previous reports and not with others [6, 7, 59]. Explanations may be found in specific patient characteristics. Most patients in our cohort had established RA with well-controlled inflammation and disease activity, showing low DAS28 and relatively preserved HGS. This may have reduced the sensitivity of DAS28 as a predictor of motor impairment in this context. While DAS28 remains a valuable clinical tool, it may not fully capture localised joint dysfunction or subtle motor deficits in well-managed RA populations. Third, the patient group that participated in the hand exercise trial was small. However, this group showed a relatively high level of compliance according to patients’ diaries. Although self-reporting can be subject to bias and may not always reflect actual adherence, it remains a practical and informative method in long-term interventions. In our study, the consistency in reported exercise over six months added credibility to the observed functional improvements, despite the limited sample size. Fourth, the intervention period may have been too short to detect a structural recovery in motor-related brain regions. While the volume increase was seen in emotion–motor integration regions (e.g., insula), changes in motor-related regions were not evident. Longer-term interventions may be needed to reliably assess plasticity in these brain areas. Fifth, the exercise programme primarily focused on improving flexibility and joint mobility, rather than strength per se. While HGS may appear more directly related to strengthening interventions, it is widely used as a proxy for overall hand function in RA [60, 61]. Given that functional impairment in RA reflects both mechanical joint restrictions and pain-related neural inhibition, flexibility-based exercises may still contribute to improved hand performance. Furthermore, HGS assessment is rapid, operator-independent, and well tolerated by patients, supporting its use as a pragmatic outcome measure in this context. However, its low specificity should be acknowledged, as it cannot distinguish between different mechanisms of functional change.

## Conclusion

Our findings suggest that reduced HGS in RA may reflect both peripheral joint pathology and variation in motor-related brain structures. Associations with frontoparietal volume point to a possible central contribution to motor impairment, beyond what is captured by joint-level pathology alone. Hand exercise was associated with improved strength and subtle changes in brain structure, but its role in modulating motor-related regions remains uncertain. These results call for a more integrated view of RA-related disability shaped by inflammation, joint destruction and brain structure. Further research is needed to clarify how persistent rehabilitation and systemic treatment might jointly support motor integrity across the brain and joint domains.

## Supplementary Information

Below is the link to the electronic supplementary material.

Supplementary Material 1: Additional file 2: Fig S1. Flowchart of participant recruitment. PDF document.

Supplementary Material 2: Additional file 3: Table S2. Associations between handgrip strength (HGS) and general health-related clinical variables. PDF document.

Supplementary Material 3: Additional file 1: Table S1. Missing data per variable. PDF document.

## Data Availability

The RNA sequencing datasets generated and analysed during the current study are available in the NCBI GEO repository with accession [GSE201670](https:/www.ncbi.nlm.nih.gov/geo/query/acc.cgi? acc=GSE201670) (CD14⁺) and [GSE201669](https:/www.ncbi.nlm.nih.gov/geo/query/acc.cgi? acc=GSE201669) (CD4⁺).Due to ethical and legal restrictions related to patient confidentiality and the handling of pseudonymised MRI data under GDPR, the imaging and associated clinical datasets are not publicly available. Data may be made available upon reasonable request to the corresponding author, subject to review of a research proposal, relevant ethical approval, and a data use agreement to ensure compliance with data protection and ethical requirements.

## Abbreviations

aCCP: Anti-cyclic citrullinated peptide antibodies
AICc: Corrected akaike information criterion
BMI: Body mass index
BG: Body mass index
CD: Cluster of differentiation
CRP: C-reactive protein
DAS28: Disease activity score, based on 28-joint count
DD: Disease duration
DASH: Disabilities of the arm, shoulder and hand questionnaire
ELISA: Enzyme-linked immunosorbent assay
ES: Erosion score
ESR: Erythrocyte sedimentation sate
FIQ: Fibromyalgia impact questionnaire
GSEA-KEGG: Gene set enrichment analysis – Kyoto encyclopedia of genes and genomes
HAQ: Health assessment questionnaire
HGS: Handgrip strength
IFG: Inferior frontal gyrus
IL: Interleukin
ITK: IL2-Inducible T Cell Kinase
JAKi: Janus Kinase Inhibitor
JSN: Joint space narrowing
LASSO: Least absolute shrinkage and selection operator
LMM: Linear mixed-effects model
LPS: Lipopolysaccharide
M1: Primary motor cortex
MRI: Magnetic resonance imaging
N: Newton
PBMC: Peripheral blood mononuclear cell
PMA/SMA: Premotor and supplementary motor area
PPC: Posterior parietal cortex
RA: Rheumatoid arthritis
S1: Primary somatosensory cortex
SBP: Systolic blood pressure
SHS: Sharp/van der Heijde Score
SJC: Swollen joint count
SMA: Supplementary motor area
STS: Study coordinator
Tha: Thalamus
TJC: Tender joint count
TNF: Tumor necrosis factor
TNFi: Tumor necrosis factor Inhibitor
TOS: Thumb opening size
