# Supplementary material for: Handgrip weakness is associated with motor cortex atrophy in rheumatoid arthritis: a cross-sectional study with a hand exercise intervention
Source: BMC Med. 2026 May 26;24:331. doi: 10.1186/s12916-026-04956-z (PMC13214063; doi:10.1186/s12916-026-04956-z)
Supplement: Supplementary file 1 — Supplementary Material 1: Additional file 2: Fig S1. Flowchart of participant recruitment. PDF document. [file 12916_2026_4956_MOESM1_ESM.pdf]

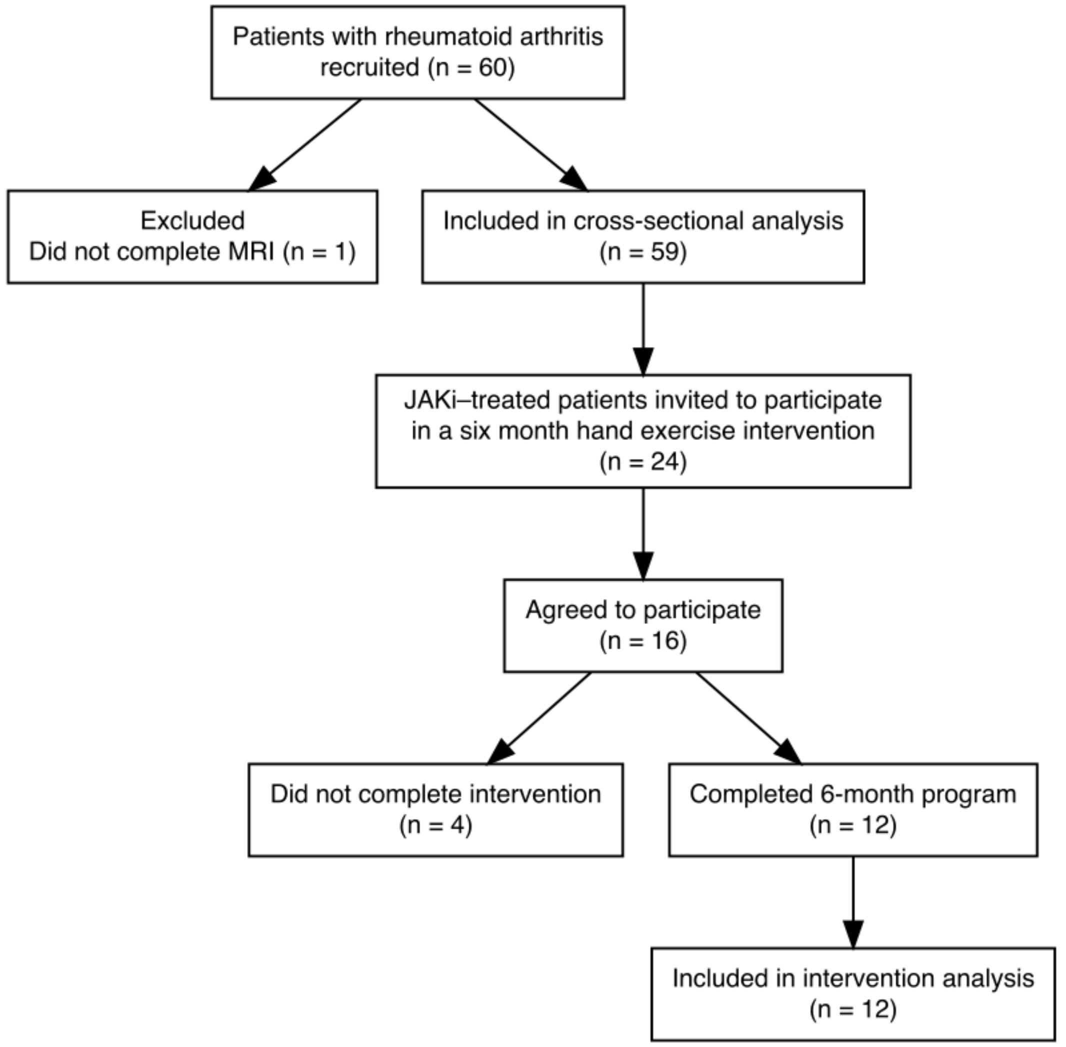

Additional file 2: Fig S1. Flowchart of study patient recruitment.

The figure illustrates the recruitment of participants, completion of baseline assessments, reasons for exclusion, and the final number of patients included in the cross-sectional analysis. It also shows the subset of patients receiving Janus kinase inhibitor (JAKi) treatment who were invited to participate in the hand exercise intervention, the number who agreed to take part, attrition during follow-up, and the final sample included in the 6-month intervention analysis.
