# Supplementary material for: Handgrip weakness is associated with motor cortex atrophy in rheumatoid arthritis: a cross-sectional study with a hand exercise intervention
Source: BMC Med. 2026 May 26;24:331. doi: 10.1186/s12916-026-04956-z (PMC13214063; doi:10.1186/s12916-026-04956-z)
Supplement: Supplementary file 2 — Supplementary Material 2: Additional file 3: Table S2. Associations between handgrip strength (HGS) and general health-related clinical variables. PDF document. [file 12916_2026_4956_MOESM2_ESM.pdf]

Additional file 3: Table S2. Associations between handgrip strength and general health-related clinical variables.

**General health variables**	**β**	**p-value**	**SE**
HAQ total	-0.54	$p < 0.0001$	0.113
FIQ total	-0.05	0.69	0.113
BMI	-0.09	0.36	0.101
SBP	0.04	0.66	0.102

β-coefficients, standard errors (SE), and p-values are derived from linear mixed-effects models with HGS as the outcome, adjusted for hand dominance and with participant ID included as a random intercept. Continuous variables were log-transformed and standardised where appropriate. Abbreviations: HAQ, Health Assessment Questionnaire; FIQ, Fibromyalgia Impact Questionnaire; BMI, body mass index; SBP, systolic blood pressure.
