# Supplementary material for: Handgrip weakness is associated with motor cortex atrophy in rheumatoid arthritis: a cross-sectional study with a hand exercise intervention
Source: BMC Med. 2026 May 26;24:331. doi: 10.1186/s12916-026-04956-z (PMC13214063; doi:10.1186/s12916-026-04956-z)
Supplement: Supplementary file 3 — Supplementary Material 3: Additional file 1: Table S1. Missing data per variable. PDF document. [file 12916_2026_4956_MOESM3_ESM.pdf]

Additional file 1: Table S1. Missing data per variable

Variables	Missing values
**Clinical variable**	
Age	0
HGS	0
Dominance	0
SHS	8 (13.6)
TJC	0
SJC	0
TOS	0
DASH (total/subscales)	0
DD	2 (3.4)
DAS28	2 (3.4)
CRP	2 (3.4)
SBP	0
BMI	0
HAQ	0
FIQ	0
**Motor-related brain region**	
M1	0
S1	0
PMA/SMA	0
IFG	0
PPC	0
BG/Tha	0
Cerebellum	0
Insula	0

Values are presented as n (%). Abbreviations: HGS, handgrip strength; TOS, thumb opening size; SHS, Sharp/van der Heijde Score; TJC, tender joint count; SJC, swollen joint count; DASH, Disabilities of the Arm, Shoulder and Hand; DD, disease duration; DAS28, Disease Activity Score in 28 joints; CRP, C-reactive protein; SBP, systolic blood pressure; BMI, body mass index; HAQ, Health Assessment Questionnaire; FIQ, Fibromyalgia Impact Questionnaire; M1, primary motor cortex; S1, primary somatosensory cortex; PMA/SMA, premotor area/supplementary motor area; IFG, inferior frontal gyrus; PPC, posterior parietal cortex; BG/Tha, basal ganglia/thalamus.
